# Incidence and treatment of hand and wrist injuries in Dutch emergency departments

**DOI:** 10.1007/s00068-021-01732-x

**Published:** 2021-07-01

**Authors:** Roderick H. van Leerdam, Pieta Krijnen, Martien J. Panneman, Inger B. Schipper

**Affiliations:** 1 Department of Trauma Surgery, Leiden University Medical Center (LUMC), Albinusdreef 2, 2333 ZA Leiden, The Netherlands; 2 Consumer Safety Institute, Amsterdam, The Netherlands

**Keywords:** Incidence, Hand, Wrist, Emergency department

## Abstract

**Purpose:**

The purpose of this study was to describe the epidemiology, treatment and costs of hand and wrist injuries presenting to the Dutch ED. With increasing medical costs and crowding of emergency departments (ED), a more detailed description of emergency department attendance of hand and wrist injuries and their treatment may help to facilitate more adequate allocation of health care services.

**Methods:**

The Dutch Injury Surveillance System obtained a total of 160,250 hand and wrist injuries. Patient characteristics, incidence rates, type of injury, treatment, and costs were described.

**Results:**

The incidence of hand and wrist injuries in the Netherlands in 2016 was 11 per 1000 in males and 8 per 1000 in females. This is about 25% of all injuries presented at the ED. Of all hand injuries, only 3% was directly admitted to the hospital or received emergency surgery. Thirty percent did not need further treatment in the hospital.

**Conclusion:**

The current data suggest that a substantial proportion of the hand and wrist injuries needed no subsequent specialized treatment. Although the severity of the injury could not be deduced from our data, the data suggest a ground for a more extensive role of primary health care (general) practitioners in the primary triage and treatment of hand and wrist injuries. This may reduce health care cost and help decongest the ED departments. Prospective studies are needed to confirm these preliminary conclusions.

**Level of evidence:**

III.

## Introduction

Injuries of the hand and wrist are among the most common injuries in all age groups and carry the risk of serious handicap [1]. With increasing medical costs and crowding of emergency departments, a more detailed description of emergency department attendance of hand and wrist injuries and their treatment may help to facilitate adequate allocation of healthcare services. Epidemiologic studies about the incidence and management of hand and wrist injuries in the Netherlands are scarce and mostly outdated, or describe only specific subgroups of injuries [2–4].

The purpose of this study was to describe the epidemiology and treatment of hand and wrist injuries presented in the Dutch Emergency Departments (ED). Furthermore, we studied the characteristics of these injuries regarding primary treating specialty and costs.

## Patients and materials

### Research questions

This descriptive study aimed to answer the following questions: (1) What are the incidence rates and distribution of different hand and wrist injuries among patients visiting the ED? (2) Can specific groups at risk for these injuries be identified? (3) How are hand and wrist injuries treated and by which specialties? (4) What are the direct medical costs of hand and wrist injuries?

### Study design, data source and study population

The data for this retrospective cohort study were extracted from the Dutch Injury Surveillance system (DISS). The registry consists of a continuous data collection from 14 Dutch hospital-based emergency departments. These EDs are regarded to be a representative sample for the total number of injured patients presenting at EDs in the Netherlands [5, 6]. The hospitals consist of three university hospitals, one non-academic teaching hospital and ten general hospitals. The hospitals participating in the DISS serve 13% of the total Dutch population of 17.02 million (2016). Injuries of all age groups are recorded. For the present study, all records of hand and wrist injuries presenting at the ED in 2016 were collected. These included all injuries to the phalanx, metacarpal, carpal, distal radius, and ulna.

To generate national estimates of hand and wrist injuries, data were extrapolated to the Dutch population. The number of ED visits registered due to hand and wrist injuries by the participating hospitals was multiplied by the quotient of the total number of ED visits in the Netherlands divided by the total number of ED visits registered in the participating hospitals [7].

### Data

Since the data in the DISS are de-identified, no institutional review board approval was required. Registered data included gender, age, and information on diagnosis, treatment, and discharge. Ages of patients were categorized in 5 year age groups.

Age and gender-specific incidence rates were calculated as the ratio of the estimated number of hand/wrist injury-related ED visits in the Netherlands and the age and gender-specific population numbers [8].

Causes of injury were recorded according to the International Classification of External Causes of Injuries divided into six categories: home and leisure, sports, occupational, self-mutilation, violence and traffic [5]. Type of injury was divided into three categories: (1) laceration/wound; (2) fractures, and (3) other injuries including sprains, contusions, tendon ruptures, amputations, burns, allergic reactions or not specified types of injuries.

Injury treatment was divided into five categories: no further treatment, referred to general practitioner, conservative/outpatient clinic, emergency operation/admission and unknown. The primary treating specialty was also recorded.

### Medical costs

Direct medical costs as a result of injury to wrist or hand were calculated with the use of the incidence-based Dutch Burden of Injury Model [9, 10]. This model calculates healthcare consumption and related costs for predefined injury patient groups that are homogenous in terms of health service use. We included all health services that are relevant for the treatment and rehabilitation of injury patients. Data on injury-related healthcare consumption were obtained from the DISS (ED visits), Hospital Discharge Registry (admissions, duration, procedures), nursing home and home care registers [8] and an injury patient follow-up survey conducted in 2007–2008 [11].

Health care costs of injuries were calculated by multiplying incidence, transition probabilities (e.g. chance of hospital admission, outpatient visit, G.P. care, home care, physical therapy), health care volumes (e.g., length of stay in hospital or institution, the number of outpatient visits, G.P. visits, home care hours, and physical therapy treatments) with unit costs (e.g., costs per day in the hospital, cost per outpatient visit, G.P. visit). All unit costs were estimated according to national guidelines for healthcare costing [12].

## Results

In 2016, 19,324 patients with hand or wrist injuries were treated in the hospitals that participated in the DISS. This number was extrapolated to 160,250 hand/wrist injuries in the Netherlands in 2016.

### Incidence

The number of hand and wrist injuries treated at the EDs in the Netherlands in 2016 was 90,620 in males (57%) and 69,630 in females. This is about 25% of all injuries that present to the ED department. The mean age of the patient population was 33 with a range from 0 to 107 years. Both sides were equally affected (49% left vs. 47% right) and bilateral injuries were uncommon (4%).

**Fig. 1:**
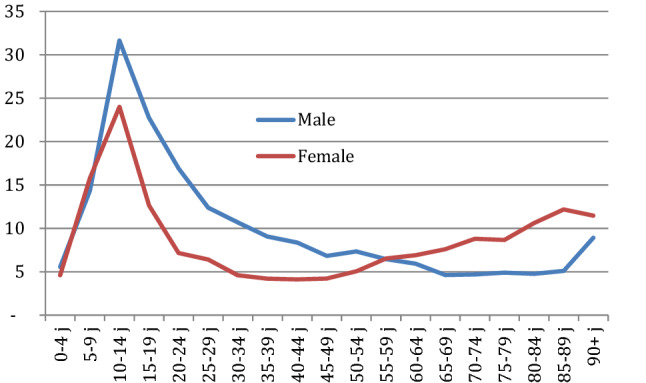
Incidence of hand and wrist injuries per 1000 inhabitants in the Netherlands in 2016, by sex and age group

The population incidence rates were 11/1000 in males and 8/1000 in females. Figure 1 shows the incidence per 1000 inhabitants per age group with a peak incidence for injuries in 10–14-year-olds. The incidence in males was higher in the ages below 55 years, while the incidence in the female population was higher after 55 years.

### Injury characteristics

Table 1 shows the incidence and treatment of hand injuries per type of injury. Most patients presented with a fracture (55%) or a laceration/wound (33%). Other injuries, such as sprains, burns or contusions, occurred in 12% of the patients.

**Table 1:** Distribution, incidence, and treatment of hand and wrist injuries presented in Dutch emergency departments in 2016, by type of injury

Injury type	Incidence per 1000	Injury type of total (%)	Treatment				
			No further treatment (%)	Referred to General Practitioner (%)	Conservative/Outpatient (%)	Emergency operation/Admission (%)	Unknown (%)
Laceration/wound	3.0	33	60	9	23	2	6
Wrist	0.6	20	68	7	19	2	5
Carpal/metacarpal	0.9	28	64	10	17	3	6
Finger	1.6	52	55	10	27	2	6
Fracture	5.2	55	12	1	82	2	3
Wrist	2.7	52	9	0	86	3	2
Carpal/metacarpal	0.8	16	11	1	85	1	3
Finger	1.7	32	17	1	76	1	4
Other	1.2	12	29	3	57	7	5
Wrist	0.2	15	45	6	40	5	3
Carpal/metacarpal	0.2	14	32	4	48	9	7
Finger	0.8	71	24	2	62	8	4
Total			30	4	60	3	4

Fractures were most often wrist fractures (52%) followed by finger fractures (32%).

After initial treatment at the ED, 30% of all injuries did not need further treatment. Three percent of all hand and wrist injuries were directly admitted or received emergency surgery. Admissions without surgery were rare yet the exact number could not be specified. About half (52%) of the injuries were caused by home and leisure accidents and about a quarter of these happened during sports activities (24%). Occupational, violence or self-mutilation injuries occurred in 14% and traffic injuries in 10% of the cases.

Primary treating specialties are summarized in Table 2. The majority of patients with hand and wrist injuries were initially treated by trauma surgeons (56%) followed by orthopedic surgeons (23%) and ED physicians (17%). Plastic surgeons were primarily involved in 2% of the patients.

**Table 2:** Primary treating specialty per type of injury

Primary treating Speciality	Laceration/wound	%	Fracture	%	Other	%	Total	%
Emergency department	12,941	25	10,674	12	3524	18	27,139	17
Trauma surgery	26,757	52	53,104	60	10,468	52	90,329	56
Orthopedics	9550	18	22,341	25	4517	23	36,408	23
Plastic surgery	1042	2	317	0.4	1083	5	2442	2
Unknown / other specialty	1459	3	2068	2	405	2	3932	2
Total	51,749	100	88,504	100	19,997	100	160,250	100

### Direct medical costs

The average direct medical costs of hand injuries were calculated to be €1500 per patient. Based on 160,250 ED visits in 2016, the total health care costs of hand and wrist injuries in the Netherlands were estimated to be €260 million, which equals 13% of the total direct medical costs made by patients presenting at Dutch ED departments in 2016 in the Netherlands (2 billion euro) [13]. Mean costs per patient were higher in older (> 65 years) men (€2270) and women (€3650) and the overall mean cost was higher in women (€1900) than in men (€1200). Fractures accounted for more than two-third (€172 million) of the total costs of hand injuries.

## Discussion

The main findings of this exploratory descriptive analysis are that most hand and wrist injuries presented at the ED are fractures, followed by lacerations and wounds. Children in the age group 10–14 years are at higher risk to sustain a hand or wrist injury. Thirty percent of the hand and wrist injuries do not need subsequent treatment or follow-up, whereas emergency admission or surgery is needed in only 3%. At the ED, the trauma surgeons predominantly provide the primary care for hand and wrist injuries. The majority of costs are due to fractures of the hand and wrist in patients of 55 years of age and older.

The calculated incidence of hand and wrist injuries was 19/1000 in the Netherlands in 2016. This number does not seem to change over time since the incidence in 2004 was calculated to be 18 per 1000 for the Netherlands by Larsen et al. [3]. The Dutch incidence falls within the incidence range of that in other European countries, which is estimated to be 15–38 per 1000 [3, 14–16]. The age-related peak rates of injury are similar to that found in other studies [15, 17, 18]. Previous studies show that the proportion of hand and wrist injuries out of all accidental injuries treated at the ED varies between 14 and 27% [19–21]. The numbers in the Netherlands fall within this range (25% on average) but can be considered relatively high.

Although injuries of the hand and wrist are common, only 3% of these injuries were directly admitted or treated surgically (emergency admission/surgery). This is concordant with previous literature [3, 16, 21]. Remarkably, 30% of all injuries, including 60% of the wounds and lacerations, did not need further treatment after initial treatment at the ED. This may imply that a general practitioner could probably have triaged and treated a subset of these patients. A shift of primary presentation from the hospital to primary health care could result in a considerable cost reduction, along with reduction of ED overcrowding and workload, and subsequent reduced ED waiting times. General practitioners are available during daytime and take shifts during the evening and night. Self-referral to the ED during these hours is common. In the Netherlands, out of hours primary care is increasingly provided by general practitioner cooperatives (GPC) and arose from the need to decrease the overcrowding of the ED. GPCs are usually situated in or near a hospital, sometimes sharing triage and diagnostic resources of the hospital. One of the objectives is to treat and triage more “self-referrals” otherwise presenting to the ED*,* decreasing emergency department use up to 22% [22]*.* The care for these patients at the GPC could be up to three times cheaper than in the ED [23].

The risk of missed diagnoses by the GP decreases when a GP has proper access to diagnostic facilities [24]. Given the increase of general practitioner cooperatives situated in or near a hospital, we expect this risk to be acceptable.

The fact that hand and wrist injuries comprise 25% of the injuries presented at the ED, 30% needed no further treatment combined with the finding that the majority are treated by trauma surgeons and ED physicians, but not by plastic surgeons, implies that many wounds did not require specialized care and were probably of minor severity. This is another good reason for reallocation of patients and care to the primary health care providers, in an attempt to optimize the quality of care and costs balance.

According to this study, hand injuries accounted for €260 million of direct medical costs. Hence, hand injuries constitute not only a substantial part of all injuries at the ED but also represent a considerable economic burden to society. Estimates of the economic costs of different acute hand and wrist injuries vary greatly depending on the study methodology, however, by any standards, these injuries should be considered a substantial burden on the individual and society [25]. The total costs are expected to be even higher if productivity loss due to work absenteeism is included in the calculation on top of the direct medical costs. In our opinion, accurate prevention starts by a deep understanding of the problem. Accurate data on these injuries is an essential starting point.

The strengths of this study include the fact that this study gives a renewed and detailed nationwide overview of all hand and wrist injuries presenting at the ED. In contrast to other studies, this study also takes costs into account and describes treatments.

The results also should be interpreted in light of their shortcomings. First, the data are from 2016, and more recent data were not available for analysis. However, but we do not expect that more recent data would change the results and conclusions of the study, since demographics and outcome have not changed, and since there have not been any policy changes of influence in our health care system during the past four years. Furthermore, although the DISS has proven to be a good tool to identify injuries at the ED [18, 26–29], the database uses estimates for incidence rates which may have led to inaccurate results. Also, the DISS registry does not include data explaining why a patient is admitted or if he is (self)-referred. Unfortunately, the database included only records on hand and wrist injuries presented at the ED. This underestimates the true nationwide incidence of hand and wrist injuries, since minor injuries are often presented to and treated by the general practitioner in the Netherlands. Also, patients that are treated in hospital in a planned fashion were not included in our study. Another limitation is that the database fails to register changes in diagnosis after the ED visit. This was also a shortcoming described by Beerekamp et al. [30], possibly causing an over- or underestimation of certain specific hand and wrist injuries. Finally, this study only estimated the direct medical costs including all health services.

In our opinion, this study provides an accurate overview of hand and wrist injuries treated at the EDs in the Netherlands. Given the crowding of the ED departments, the current data suggests that a substantial proportion of the hand and wrist injuries needed no subsequent specialized treatment. Although the severity of the injury could not be deduced from our data, the data suggest a ground for a more extensive role of primary health care (general) practitioners in the primary triage and treatment of hand and wrist injuries, provided that general practitioners are sufficiently equipped with additional education, diagnostic facilities and surgical expertise. This may reduce health care cost and help decongest the ED departments. Prospective studies are needed to confirm these preliminary conclusions.

## Data Availability

The data that support the findings of this study are available from the Dutch Injury Surveillance system (DISS). Restrictions apply to the availability of these data, which were used under license for this study. Data are available Martien Pannenman with the permission of DISS.

## Code availability

All code for data cleaning and analysis associated with the current submission is available through Martien Pannenman.
